# Metabolic Syndrome in Fasting and Non-Fasting Participants: The UAE Healthy Future Study

**DOI:** 10.3390/ijerph192113757

**Published:** 2022-10-22

**Authors:** Fatima Mezhal, Amar Ahmad, Abdishakur Abdulle, Andrea Leinberger-Jabari, Abderrahim Oulhaj, Abdulla AlJunaibi, Abdulla Alnaeemi, Ayesha S. Al Dhaheri, Eiman AlZaabi, Fatma Al-Maskari, Fatme AlAnouti, Habiba Alsafar, Juma Alkaabi, Laila Abdel Wareth, Mai Aljaber, Marina Kazim, Manal Alblooshi, Mohammad Al-Houqani, Mohammad Hag Ali, Naima Oumeziane, Omar El-Shahawy, Rami H. Al-Rifai, Scott Sherman, Syed M. Shah, Tom Loney, Wael Almahmeed, Youssef Idaghdour, Luai A. Ahmed, Raghib Ali

**Affiliations:** 1 Public Health Research Center, New York University Abu Dhabi, Abu Dhabi 129188, United Arab Emirates; 2 Department of Epidemiology and Public Health, College of Medicine and Health Sciences, Khalifa University, Abu Dhabi 127788, United Arab Emirates; 3 Department of Pediatrics, Zayed Military Hospital, Abu Dhabi 72763, United Arab Emirates; 4 Department of Cardiology, Zayed Military Hospital, Abu Dhabi 72763, United Arab Emirates; 5 Department of Nutrition and Health, College of Medicine and Health Sciences, UAE University, Al-Ain 15551, United Arab Emirates; 6 Department of Pathology, Sheikh Shakhbout Medical City, Abu Dhabi 11001, United Arab Emirates; 7 Institute of Public Health, College of Medicine and Health Sciences, UAE University, Al-Ain 15551, United Arab Emirates; 8 Zayed Center for Health Sciences, UAE University, Al-Ain 15551, United Arab Emirates; 9 College of Natural and Health Sciences, Zayed University, Abu Dhabi 144534, United Arab Emirates; 10 Center for Biotechnology, Khalifa University, Abu Dhabi 127788, United Arab Emirates; 11 Department of Genetics and Molecular Biology, Khalifa University, Abu Dhabi 127788, United Arab Emirates; 12 Department of Biomedical Engineering, Khalifa University, Abu Dhabi 127788, United Arab Emirates; 13 Department of Internal Medicine, College of Medicine and Health Sciences, UAE University, Al-Ain 15551, United Arab Emirates; 14 Pathology and Laboratory Medicine Institute, Cleveland Clinic Abu Dhabi, Abu Dhabi 112412, United Arab Emirates; 15 Healthpoint Hospital, Abu Dhabi 112308, United Arab Emirates; 16 Abu Dhabi Blood Bank Services, SEHA, Abu Dhabi 109090, United Arab Emirates; 17 Department of Medicine, College of Medicine and Health Sciences, UAE University, Al-Ain 15551, United Arab Emirates; 18 Department of Health Science, Higher Colleges of Technology, Abu Dhabi 25026, United Arab Emirates; 19 Department of Population Health, New York University School of Medicine, New York, NY 10595, USA; 20 College of Medicine, Mohammed Bin Rashid University of Medicine and Health Sciences, Dubai 505055, United Arab Emirates; 21 Heart and Vascular Institute, Cleveland Clinic Abu Dhabi, Abu Dhabi 112412, United Arab Emirates; 22 MRC Epidemiology Unit, University of Cambridge, Cambridge CB2 0SL, UK

**Keywords:** metabolic syndrome, central obesity, diabetes, hypertension, dyslipidemia, United Arab Emirates

## Abstract

Introduction: Metabolic syndrome (MetS) is a multiplex of risk factors that predispose people to the development of diabetes and cardiovascular disease (CVD), two of the major non-communicable diseases that contribute to mortality in the United Arab Emirates (UAE). MetS guidelines require the testing of fasting samples, but there are evidence-based suggestions that non-fasting samples are also reliable for CVD-related screening measures. In this study, we aimed to estimate MetS and its components in a sample of young Emiratis using HbA1c as another glycemic marker. We also aimed to estimate the associations of some known CVD risk factors with MetS in our population. Methods: The study was based on a cross-sectional analysis of baseline data of 5161 participants from the UAE Healthy Future Study (UAEHFS). MetS was identified using the NCEP ATP III criteria, with the addition of HbA1c as another glycemic indicator. Fasting blood glucose (FBG) and HbA1c were used either individually or combined to identify the glycemic component of MetS, based on the fasting status. Multivariate regression analysis was used to test for associations of selected social and behavioral factors with MetS. Results: Our sample included 3196 men and 1965 women below the age of 40 years. Only about 21% of the sample were fasting at the time of recruitment. The age-adjusted prevalence of MetS was estimated as 22.7% in males and 12.5% in females. MetS prevalence was not statistically different after substituting FBG by HbA1c in the fasting groups (*p* > 0.05). Age, increased body mass index (BMI), and family history of any metabolic abnormality and/or heart disease were consistently strongly associated with MetS. Conclusion: MetS is highly prevalent in our sample of young Emirati adults. Our data showed that HbA1c may be an acceptable tool to test for the glycemic component of MetS in non-fasting samples. We found that the most relevant risk factors for predicting the prevalence of MetS were age, BMI, and family history.

## 1. Introduction

Metabolic syndrome (MetS) refers to the multifactorial clustering of metabolic and pathophysiological cardiovascular risk factors, such as obesity, dyslipidemia, hypertension, and hyperglycemia [1]. The combination of these components places individuals at high risk for developing Type 2 diabetes (T2D) and cardiovascular disease (CVD), the two major non-communicable diseases (NCDs) that account for 45% of all deaths in the United Arab Emirates (UAE) [2,3].

Each of the MetS components is an independent risk factor for CVD and is highly prevalent in the UAE, as described in previous studies [4,5]. The accumulation of these risk factors elevates the rate and severity of CVD [6]. A meta-analysis of 950,000 participants showed that MetS was associated with a 2-fold increase in CVD risk and CVD-related mortality, myocardial infarction, and stroke [7]. Several longitudinal studies also showed the association between MetS and CVD events [8,9].

MetS became a topic of focus in the past two decades as it became more prevalent in the general population. Globally, MetS prevalence exceeds 20% in adults [10]. In the UAE, a recent study by Nabil et al. [11] on participants from Sharjah and the Northern Emirates estimated MetS prevalence as high as 33.6% in Emiratis. 

MetS is traditionally assessed using the National Cholesterol Education Program Adult Treatment Panel-III (NCEP ATP III) guideline [12]. The guideline provides cut-off values for the five MetS components, two of which are physical measurements and the other three are from fasting blood samples. Because obtaining fasting samples are not always feasible for screening purposes, especially in opportunistic research sampling settings, researchers are now advising to use non-fasting blood samples as screening tools [13,14]. Advocates of the non-fasting samples point out that they can still provide a high degree of accuracy in CVD risk identification and should become standard for screening [15].

The main objective of the study is to estimate the prevalence of MetS and its components in Emiratis below 40 years, using fasting and non-fasting samples, by introducing the hemoglobin A1c (HbA1c) as an additional glycemic marker. We also assess the association of some known CVD risk factors with MetS in our population. 

## 2. Methods 

### 2.1. Study Sample

The study subjects were Emirati adults taking part in the UAE Healthy Future Study (UAEHFS) [16]. The study was based on a cross-sectional analysis of the baseline data collected between February 2016 to December 2018. Eligibility criteria were Emiratis aged 18 to 40 years without acute illnesses or pregnancy. All participants provided informed consent. This study followed the principles outlined in the Declaration of Helsinki and was approved by the Abu Dhabi Health Research and Technology Committee (ref. DOH/HQD/2020/516). Additional information on the UAEHFS methodology is published elsewhere [16].

### 2.2. Data Collection

The UAEHFS collects participant data by employing three steps. First, a self-completed questionnaire that collected sociodemographic data as well as data on health status; smoking; family history of heart disease and stroke; and NCDs including obesity, diabetes, high cholesterol, and hypertension. Participants then underwent physical measurements including three repeat measures of brachial blood pressure and anthropometrics (weight, height, waist and hip circumferences). Finally, a blood sample was collected to measure glycemic and lipid panels.

### 2.3. Metabolic Syndrome Criteria

In this study, we used the NCEP ATP III criteria to identify MetS. According to this definition, MetS is present if three or more of the following criteria are met: waist circumference ≥ 102 cm in men and ≥88 cm in women, blood pressure measurements ≥ 130/85 mmHg or taking blood pressure medication, fasting triglycerides (TG) level ≥ 150 mg/dL or taking cholesterol-controlling medication, fasting high-density lipoprotein (HDL) cholesterol <40 mg/dL in men, <50 mg/dL in women or taking cholesterol-controlling medication, and fasting blood glucose (FBG) ≥ 100 mg/dL or taking anti-diabetic medication. 

For non-fasting samples, we have introduced another cut-off for TG set at ≥175 mg/dL as recommended by the joined consensus initiative of the European Atherosclerosis Society and the European Federation of Clinical Chemistry and Laboratory Medicine [14,17,18].

Because blood glucose has been purported to only be reliable in fasting samples, we included glycated hemoglobin A1C (HbA1c) as an additional glycemic indicator in our study. We used the standardized cut-off of HbA1c ≥ 5.7 to additionally identify hyperglycemia in the sample [19].

## 3. Statistical Analyses

Baseline characteristics of the study participants were presented by sex. Continuous variables were presented as means ± standard deviation, and categorical data were presented as frequencies and percentages. For the continuous variables, differences in means were measured by Welch *t*-tests, while for frequencies and percentages, the differences in distribution between groups were tested using the chi-square test. Age-adjusted prevalence was estimated using logistic models and presented with a corresponding 95% confidence interval (CI).

Three multivariate logistic regression models were performed with MetS (yes = 1 vs. no = 0) as an outcome using FBG, HbA1c, and FBG and/or HbA1c as one criterion of MetS. The independent variables were age (years); BMI category (overweight/obese); smoking (yes/no); family history of heart disease or metabolic abnormality (yes/no); and social determinants, specifically employment status (employed/unemployed/student), education level (higher education/lower education), and marital status (married/not married). Odds ratios (ORs) with corresponding 95% CIs as well as z-values (*p*-values) were reported. The likelihood ratio chi-square test was reported with corresponding degrees of freedom and *p*-value for each fitted logistic regression model. To assess the performance of each fitted regression model, the area under the ROC curve (AUC) was used as a measure of discrimination. The corresponding lower and upper 95% CI of the AUC were computed. All statistical analyses were conducted by subgroup analysis based on gender (females and males) as well as fasting status.

Statistical analyses were performed in Stata 15 software [20]. All applied tests were two-sided and a *p*-value < 0.05 was accepted as statistically significant. No *p*-value adjustment was performed for multiple comparisons. Missing data were categorized as a group of unknowns and those unknown missing value groups were included in the analyses.

## 4. Results

A total of 5161 participants (62% male) aged between 18 and 40 years were included in the study. The mean age (±SD) of the study population was 25.7 (±6.2) years; by gender, the mean ages were 26.4 (±5.9) years in men and 24.5 (±6.3) years in women (*p* < 0.001). 

Table 1 presents the previous diagnosis of (or treatment for) chronic disease, behavioral risk factors, and family history of metabolic disease or heart disease. Self-reported diabetes or taking antidiabetic medication was reported for 3.1% of the total cohort, 6.6% reported hypertension, and 11.6% reported hyperlipidemia. Current smoking was reported in 38.1% of men and only 4.1% of women. Family history of any metabolic abnormality and/or heart disease was reported in 52.4% of men and 58.7% of women. Social determinants such as employment status, highest level of education, and marital situation are also described in Table 1. 

Approximately 21% of participants were fasting, 24.3% of men and 15.5% of women. Because metabolic syndrome (MetS) is usually tested using fasting samples, the prevalence of MetS and its biomarkers was reported in fasting and non-fasting groups for men and women in Table 2 and Table 3, respectively. Comparative analysis of the fasting and non-fasting populations is described in Supplementary Table S1.

In men, MetS components were similar between fasting and non-fasting groups, with the exception of low HDL and high triglycerides, which were significantly higher in the non-fasting group (*p* < 0.05). MetS prevalence in the fasting group was estimated using 2 models, by FBG (24.9% (95% CI 21.8–28.0%)), and by HbA1c (22.8% (95% CI 19.8–25.9%)). MetS prevalence did not significantly change across the 2 models. The combination of both glycemic markers gave a higher prevalence (26.0% (95% CI 22.9–29.2%)), but it was not statistically different from using each glycemic marker alone (*p* > 0.05). There was a high agreement between the MetS-by FBG and the MetS-by HbA1c models, estimated as 95.6% (Kappa = 0.882). The area under the curve of the models and agreement rates are summarized in Table 4. Testing MetS by HbA1c were similar in fasting and non-fasting groups. The overall prevalence of MetS in the male population using both glycemic markers interchangeably resulted in 22.7% (95% CI 21.2–24.2%).

In women, central obesity and low HDL were significantly higher in the fasting group (*p* < 0.05). MetS by FBG alone resulted in 16.6% (95% CI 12.1–21.1%) and by HbA1c in 17.7% (95% CI 13.1–22.2%) in fasting women, and they did not significantly change across the 2 models. Combining both glycemic markers resulted in 18.0% (95% CI 13.4–22.6%) in fasting women, but it was similar to MetS by the other models (*p* > 0.05). The agreement between the 3 models was above 98.0% (Kappa > 0.940). In women, MetS by HbA1c was significantly higher in fasting than non-fasting (17.7 (95% CI 13.1–22.2%) vs. 11.5 (95% CI 9.9–13.2%) respectively). The overall prevalence in the female population using both glycemic markers interchangeably resulted in 12.5% (95% CI 11.0–14.0%). 

**Figure 1:**
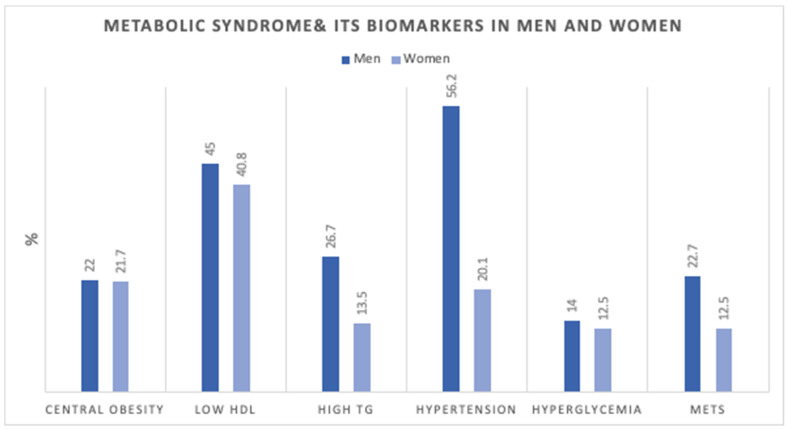
Age-adjusted Prevalence (%) of metabolic syndrome components in men (*n* = 3196) and women (*n* = 19,651). HDL; high-density lipoprotein, TG; triglycerides, MetS; Metabolic Syndrome.

**Figure 2:**
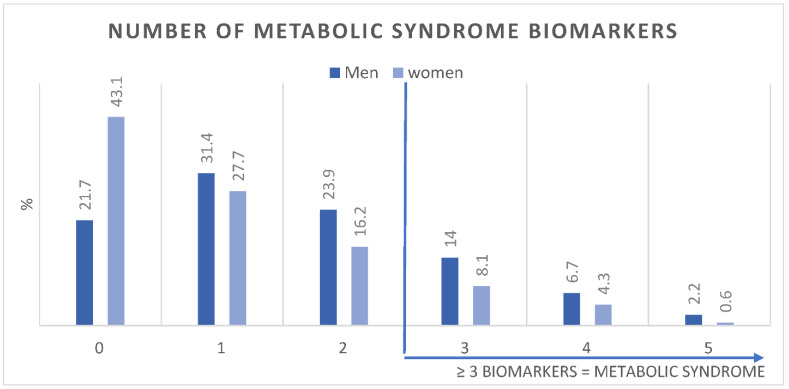
Age-adjusted Prevalence (%) of number of metabolic syndrome biomarkers in men (*n* = 3196) and women (*n* = 19,651).

Figure 1 shows the age-adjusted prevalence of MetS components in men and women. Figure 2 shows the number of MetS biomarkers distribution and accumulation in men and women.

Multivariate logistic regression analyses were used to test the association of specific determinants with having MetS in men and women, as presented in Table 5 and Table 6, respectively. Age, increased BMI, and family history of heart disease and/or any metabolic abnormality were found consistently associated with an increase in the odds of having MetS in both men and women.

## 5. Discussion

This study showed the prevalence of MetS and its components in a large sample of young Emirati adults. In addition, it also showed for the first time in the region, the capability of HbA1c, as a substitute for- or in adjunct to FBG, to estimate MetS. Since the NCEP ATP III defines MetS using FBG as one of the five components, we introduced HbA1c ≥ 5.7 as an additional glycemic indicator to cater to the 80% of the sample that were not fasting. In this cross-sectional analysis of young Emiratis, we found that MetS components were highly prevalent. Accumulating 3 or more of the components was identified as having MetS; which was prevalent in 22.7% of men and 12.5% of women. 

Our study identified differences in MetS components between men and women. It was shown that hypertension and low HDL were the most prevalent in men, while low HDL and central obesity were highest in women. Similar to a recent study on MetS in Northern cities in the UAE [11], raised blood pressure was the most prevalent MetS component among men, and among women the most prevalent components were central obesity, low HDL, followed by raised blood pressure. In their study, the MetS accounted for 33.6% in the Emirati population. The lower prevalence we showed can be explained by the younger age groups included in our study compared to theirs. 

In fasting men, we found that MetS by FBG alone was higher (but not statistically significant) than MetS by HbA1c, 24.9 (95% CI 21.8–28.0) and 22.8% (95% CI 19.8–25.9), respectively. Furthermore, MetS by the combination of both tests resulted in a 1.4% increase in the prevalence, 26.0% (95% CI 22.9–29.2). The agreement of MetS-by FBG with MetS-by HbA1c was 95.6% with a kappa coefficient of 0.882 (Table 4). MetS-by HbA1c in fasting and non-fasting groups were not statistically different, although triglycerides and HDL were significantly higher in the non-fasting sample. The combination of fasting and non-fasting groups using both glycemic indicators resulted in an overall prevalence of 22.7% in the male sample. 

In women, MetS in the fasting group was estimated as 16.6% (95% CI 12.1–21.1) by FBG alone. Substituting FBG with HbA1c increased the prevalence to 17.7% (95% CI 13.1–22.2), and combining both glycemic indicators as one biomarker further increased the prevalence to 18% (13.4–22.6). The agreement rate of MetS-by FBG and MetS-HbA1c was 98.36% with a Kappa coefficient of 0.9471. Using HbA1c to estimate MetS in the non-fasting group resulted in 11.5% (95% CI 9.9–13.2) prevalence. We believe that this significant reduction of MetS in the non-fasting group could be attributable to the differences in the fasting and non-fasting groups such as the higher waist circumference (cm) and low HDL (mg/dL) (*p* < 0.05), rather than the HbA1c % per se, as the mean of HbA1c was not statistically different in both groups (mean values in fasting and non-fasting groups are presented in Supplementary Table S1). The combination of fasting and non-fasting groups using both glycemic markers resulted in an overall prevalence of 12.5% in the female sample. 

There are several studies performed worldwide that tested the effectiveness of using HbA1c in MetS identification, as an adjunct or substitute test to FBG. Some studies showed that HbA1c helped capture more MetS cases than fasting glucose alone. Two large studies in Korea [21,22] showed that HbA1c increased the MetS prevalence from 8.5% to 10.9% and concluded that it be used as a diagnostic criterion for MetS instead of FBG. Other studies on European populations [23,24] found that substituting FBG with HbA1c also increased the MetS prevalence significantly by 4%. 

A study in Ghana [25] reported that MetS using FBG as their glycemic indicator resulted in a prevalence of 37.1%, while it increased to 52.7% when substituting FBG by HbA1C. In this study, FBG and HbA1c had good agreement using the NCEP ATP III criteria with a kappa coefficient of 0.694.

In contrast, a study in the US [26] showed that MetS using HbA1c was lower than that using FBG. However, the use of HbA1c alone resulted in a significant association with cardiovascular diseases (odds ratio 1.45). Furthermore, a study in Iran [27] also reported a lower prevalence using HbA1c versus fasting glucose: 28.6% vs. 33.5% respectively, although combining the two tools increased the total prevalence to 36.7%. The study authors concluded that HbA1c can be an acceptable surrogate for FBG. 

In addition to being tightly associated with diabetes, HbA1c has also been identified as a predictor of cardiovascular risk in non-diabetic individuals independent of FBG [28]. HbA1c has been considered a preferable tool over FBG, since it does not require a fasted state to be tested. In a study that assessed adding HbA1c as a glycemic marker, it was found that HbA1c was more closely associated with vascular health parameters including pulse wave velocity, intima media thickness, and albumin-to-creatinine ratio [29]. Therefore, HbA1c rather than FBG has better accuracy in classifying patients with cardiovascular and metabolic risk. The study authors advised that adjusting the definition of MetS by introducing HbA1c would substantially improve the accuracy of the definition and its early diagnosis. In another study exploring MetS in young Emirati female college students, HbA1c was found to be highly associated with MetS prevalence [30]. They found that an HbA1c between 5.6–6.4% increased the odds for MetS by 8.92 (95% CI 3.39–23.48), and HbA1c ≥ 6.5% increased the odds of MetS by 22.5 (95% CI 6.37–79.42). 

Growing evidence from numerous epidemiologic studies has indicated that postprandial hyperglycemia commonly precedes fasting hyperglycemia in the transition from normal glucose tolerance to overt diabetes [13,31,32]. Moreover, postprandial hyperglycemia contributes to the level of HbA1c more than fasting hyperglycemia does as HbA1c level increases through the normal range [10]. These suggest that postprandial blood sample and HbA1c levels can be reliable tools for testing for CVD risk factors, such as MetS. 

The multivariate regression analyses showed that age, increased BMI. and a positive family history of CVD risk factors were consistently associated with MetS. Our data showed that with every 1-year increase in age, the odds of having MetS increased by 5-8%. Aging is a well-recognized major risk factor for MetS and its components, and CVD risk [33]. 

It was expected that BMI would have a significant association with MetS, as it is highly correlated with waist circumference (Pearson’s correlation coefficient = 0.85). Increased BMI due to excess adipose tissue has been identified as an independent metabolic CVD risk factor [34]. Obesity is highly associated with increased central adiposity, hypertension, dyslipidemia, and glucose intolerance, all of which are components of MetS [35]. The study by Al Dhaheri et al. [30] showed that the odds of MetS increased by 3.8 and 11.2 folds in overweight and obese groups, respectively. Another study [11] also showed a significant association with increased BMI, where it increased the odds of MetS up to 1.91 (95% CI 1.25–2.91) in Emiratis.

A positive family history in our analysis included reporting parental history of heart disease, stroke, diabetes, hypertension, high cholesterol, and/or obesity. Around 53% of men and 60% of women reported having at least one of these risk factors, and our analysis showed that it increases the odds of having MetS by up to 70% in men, and by 3-folds in women. The KNHANES group [36] studied the effect of having a family history of diabetes with MetS and some of its components. They showed that individuals with a positive family history of diabetes in a first-degree relative had significantly higher rates of impaired fasting glucose and T2D. They also reported that all MetS (except for low HDL) was higher in those with a family history of T2D compared with those without. Another Sri Lankan study [37] showed the effect of having a parental history of hypertension and reported that it is positively associated with an individual’s hypertension, obesity, central obesity, and MetS. In addition, Pontiroli et al. [38] demonstrated that higher blood pressure was common in siblings of parents with T2D.

A UAE-based study published in 2020 [39] explored the association of multiple genetic risk factors to MetS. They found significant associations of multiple genetic variants with MetS. Similarly, our study can be further explored to assess the genetic predisposition for metabolic disease in a larger sample.

### Strengths and Limitations

The main strength of this study is the large sample size. This study focused on young adults below 40 years, who are often underrepresented in the context of non-communicable disease studies and metabolic syndrome. MetS was defined using a well-known guideline that employed included objective and subjective measures for more concise disease-definition criteria. Blood samples and physical measurements were collected using a standardized procedure to ensure consistent quality and reduce the risk of information bias. Another strength of the study was introducing HbA1c as an additional glycemic factor. We found that HbA1c can be used reliably in adjunct to, or substitute for FBG when a fasting sample is inconvenient to collect. We also explored the effect of other known risk factors on MetS that were not reported before. 

Most observational studies are prone to some degree of selection bias that can affect the external validity of the study. The main weakness of this study is that it is based on the voluntary recruitment of participants into the UAEHFS cohort, which therefore potentially affects the representativeness of the study sample. Additionally, the cross-sectional design of this study does not allow for inferring causality. Other limitations include the lack of physical activity and dietary data as other behavioral factors that are known to affect metabolic components, and therefore MetS.

## 6. Conclusions

MetS and its components are highly prevalent in the UAEHFS’s young Emirati population and are more prevalent in men than in women. Adding HbA1c as a glycemic indicator in adjunct to FBG can capture more MetS cases and can be used as a surrogate to FBG in non-fasting samples. We found that the most relevant risk factors for predicting the prevalence of MetS were age, increased BMI, and family history. Therefore, individuals with increased BMI and a family history of heart cardiometabolic disease can be targeted for early interventional measures.

## Figures and Tables

**Table 1:** General characteristics of the UAEHFS population (*n* = 5161).

	Men *n* = 3196 (61.9%)	Women *n* = 1965 (38.1%)
Mean (SD)		
Age, years	26.4 (5.9)	24.5 (6.3)
BMI, kg/m^2^	27.7 (6.0)	25.8 (6.6)
WC, cm	91.4 (14.5)	78.4 (14.0)
SBP, mmHg	131.2 (13.1)	117.8 (11.6)
DBP, mmHg	80.3 (10.2)	74.3 (8.6)
HDL-C, mg/dL	43.9 (10.6)	55.7 (13.0)
TG, mg/dL	118.8 (86.0)	79.3 (52.8)
fasting glucose, mg/dL	96.4 (25.7)	88.8 (22.3)
HbA1c, %	5.3 (0.7)	5.2 (0.6)
Self-reported history and/or treatment for, *N* (%)		
Diabetes mellitus	96 (3.0)	64 (3.3)
Hypertension	240 (7.5)	99 (5.0)
High cholesterol	399 (12.5)	198 (10.1)
Smoking status, *N* (%)		
Non smoker	1167 (36.5)	1458 (74.2)
Smoker	1216 (38.1)	80 (4.1)
Family history of heart disease or Metabolic abnormality, *N* (%)		
No	1456 (45.6)	769 (39.1)
Yes	1674 (52.4)	1153 (58.7)
SES determinants, *N* (%)		
Employed	1533 (48.0)	443 (22.5)
College graduate	1187 (37.1)	795 (40.5)
Married	1143 (35.8)	353 (18.0)

Abbreviations: BMI; Body Mass Index, WC; waist circumference, SBP; systolic blood pressure, DBP; diastolic blood pressure, HDL-C; High-density lipoprotein cholesterol, TG; Triglycerides, HbA1c; hemoglobin A1C, SES; socioeconomic status. Employed as opposed to unemployed or current student. College graduate opposed to having high school diploma or less. Married as opposed to being single or divorced/widowed.

**Table 2:** Age-adjusted prevalence of Metabolic syndrome and its biomarkers in Men.

	Fasting *n* = 776 (24.3%)	Non-Fasting *n* = 2420 (75.7%)	*p* Value	Total Men *n* = 3196
Central obesity (WC ≥ 102 cm)	21.6 (18.7–24.6)	22.1 (20.3–23.9)	0.554	22.0 (20.5–23.6)
Low HDL (HDL-C < 40 mg/dL *)	41.4 (37.9–45.0)	46.1 (44.1–48.2)	0.024	45.0 (43.2–46.7)
High TG (≥150 mg/dL for fasting, ≥175 mg/dL for non-fasting *)	22.2 (19.2–25.2)	28.3 (26.4–30.2)	<0.001	26.7 (25.1–28.3)
High blood pressure (≥130/85 mmHg *)	54.8 (51.2–58.3)	56.8 (54.7–58.8)	0.331	56.2 (54.4–58.0)
High FBG (≥100 mg/dL *)	25.1 (22.0–28.2)	-		8.3 (7.3–9.2)
High HbA1c (≥5.7% *)	10.1 (7.9–12.4)	8.7 (7.4–9.9)	0.266	9.1 (8.1–10.2)
Hyperglycemia (high FBG & HbA1c *)	28.7 (25.5–32.0)			14.0 (12.7–15.2)
Metabolic Syndrome (MetS):				
MetS (by FBG)	24.9 (21.8–28.0) ^‡^	-		-
MetS (by HbA1c substituting FBG)	22.8 (19.8–25.9) ^‡^	21.5 (19.8–23.2)	0.765	21.9 (20.4–23.4)
MetS (by FBG &/or HbA1c)	26.0 (22.9–29.2) ^‡^	-		22.7 (21.2–24.2)

Data are presented as percentage (confidence interval). * Includes self-report/taking controlling medication for that condition (dyslipidemia, hypertension or diabetes). ^‡^ Difference is not significant across subgroups (*p* > 0.05). WC; waist circumference, HDL; high-density lipoprotein, TG; triglycerides, FBG; fasting blood glucose, HbA1c; Hemoglobin A1C, MetS; Metabolic Syndrome. MetS is identified as having 3 out of 5 criteria. *p* value is derived from Pearson’s chi-square test for the difference between fasting and non-fasting groups.

**Table 3:** Age-adjusted prevalence of metabolic syndrome and its biomarkers in women.

	Fasting *n* = 304 (15.5%)	Non-Fasting *n* = 1661 (84.5%)	*p* Value	Total Women *n* = 1965
Central obesity (WC ≥ 88 cm)	26.4 (21.2–31.6)	20.8 (18.7–22.9)	0.037	21.7 (19.7–23.6)
Low HDL (HDL-C < 50 mg/dL *)	48.8 (43.1–54.5)	39.3 (37.0–41.8)	0.003	40.8 (38.6–43.0)
High TG (≥150 mg/dL for fasting, ≥175 mg/dL for non-fasting *)	15.9 (11.5–20.3)	13.0 (11.3–14.7)	0.081	13.5 (11.9–15.1)
High blood pressure (≥130/85 mmHg *)	20.1 (15.4–24.9)	20.1 (18.1–22.1)	0.838	20.1 (18.3–22.0)
High FBG (≥100 mg/dL *)	11.9 (8.0–15.7)	-		4.0 (3.1–4.9)
High HbA1c (≥5.7% *)	10.2 (6.6–13.8)	7.2 (5.8–8.5)	0.828	7.6 (6.4–8.9)
Hyperglycemia (high FBG/HbA1C *)	15.8 (11.5–20.1)			12.5 (11.0–14.0)
MetS (by FBG)	16.6 (12.1–21.1) ^‡^	-		-
MetS (by HbA1c)	17.7 (13.1–22.2) ^‡^	11.5 (9.9–13.2)	0.001	12.4 (10.9–14.0)
MetS (by FBG &/or HbA1c)	18.0 (13.4–22.6) ^‡^			12.5 (11.0–14.0)

Data is presented as percentage (confidence interval). * Includes self-report/taking controlling medication for that condition (dyslipidemia, hypertension or diabetes). ^‡^ Difference is not significant across subgroups (*p* > 0.05). WC; waist circumference, HDL; high-density lipoprotein, TG; triglycerides, FBG; fasting blood glucose, HbA1c; Hemoglobin A1C, MetS; Metabolic Syndrome. MetS is identified as having 3 out of 5 criteria. *p* value is derived from Pearson’s chi-square test for the difference between fasting and non-fasting proportions.

**Table 4:** The AUC and agreement rates of MetS using FBG, HbA1C, or both in the fasting sample.

		MetS (Based on FBG)	MetS (Based on HBA1C)	MetS (FBG & HBA1C)
Men (*n* = 776)	AUC	0.813 (0.780–0.847)	0.828 (0.794–0.861)	0.819 (0.786–0.851)
	LR χ^2^ (d.f.,p)	190.9 (13, <0.0001)	202.1 (13, <0.0001)	205.1 (13, <0.0001)
	Agreement%	95.62%		98.84%
	Κ (CI)	0.882		0.970 (0.900–1.00)
Women (*n* = 304)	AUC	0.853 (0.802–0.904)	0.862 (0.816–0.909)	0.860 (0.814–0.906)
	LR χ^2^ (d.f.,p)	80.6 (13, <0.0001)	86.9 (13, <0.0001)	87.2 (13, <0.0001)
	Agreement%	98.36%		98.68%
	Κ (CI)	0.947		0.958 (0.846–1.00)

AUC; Area Under the curve, LR χ^2^; Likelihood Ratio chi-square, Kappa coefficient (confidence interval). The agreement rates and Kappa coefficients are comparing MetS by FBG against MetS by HbA1c, then against MetS by FBG & HbA1c.

**Table 5:** Multivariate logistic regression analysis (Men).

	MetS in Fasting Group by FBG & HbA1c		MetS in Non-Fasting by HbA1c		MetS in Total Sample by FBG &/Or HbA1c	
	OR 95% (CI)	*p* Value	OR 95% (CI)	*p* Value	OR 95% (CI)	*p* Value
Age	1.06 (1.01–1.11)	0.027	1.06 (1.03–1.08)	<0.001	1.05 (1.03–1.07)	<0.001
BMI-overweight	3.87 (2.18–6.88)	<0.001	3.43 (2.36–5.00)	<0.001	3.50 (2.56–4.79)	<0.001
BMI-obese	17.48 (10.2–29.9)	<0.001	16.3 (11.29–24.46)	<0.001	16.71 (12.40–22.6)	<0.001
Family history	1.70 (1.11–2.61)	0.015	1.34 (1.06–1.69)	0.014	1.39 (1.14–1.69)	0.001
Smoking	1.38 (0.91–2.09)	0.131	1.12 (0.87–1.44)	0.386	1.19 (0.96–1.47)	0.123
Employment:						
Unemployed	1.13 (0.60–2.11)	0.704	0.90 (0.55–1.45)	0.660	0.98 (0.67–1.43)	0.913
Student	1.72 (0.86–3.44)	0.127	0.87 (0.56–1.35)	0.531	0.99 (0.69–1.44)	0.991
Lower education	1.04 (0.69–1.56)	0.858	1.08 (0.83–1.39)	0.573	1.03 (0.83–1.28)	0.772
Being married	1.07 (0.64–1.77)	0.796	1.20 (0.87–1.64)	0.259	1.17 (0.90–1.52)	0.253

Data is presented as Odd Ratios (95% confidence interval). Estimated values are derived from multivariate regression model including the risk factors presented in the table. Reference groups in the models are: normal BMI, no family history, non-smokers, being employed, higher education level, and unmarried. No significant interaction was detected between age and BMI.

**Table 6:** Multivariate logistic regression analysis (women).

	MetS in Fasting Group by FBG & HbA1c		MetS in Non-Fasting by HbA1c		MetS in Total Sample by FBG &/Or HbA1c	
	OR (95% CI)	*p* Value	OR 95% (CI)	*p* Value	OR 95% (CI)	*p* Value
Age	1.08 (1.00–1.15)	0.042	1.06 (1.03–1.10)	0.001	1.06 (1.03–1.10)	<0.001
BMI-overweight	4.60 (1.79–11.8)	0.002	2.51 (1.43–4.41)	0.001	2.94 (1.83–4.72)	<0.001
BMI-obese	15.20 (5.91–39.1)	<0.001	23.74 (14.77–38.16)	<0.001	20.14 (13.31–30.48)	<0.001
Family history	3.85 (1.32–11.17)	0.013	1.03 (0.70–1.50)	0.894	1.27 (0.90–1.78)	0.170
Smoking	0.62 (10.10–3.85)	0.611	0.79 (0.36–1.76)	0.571	0.73 (0.35–1.50)	0.390
Employment:						
Unemployed	1.63 (0.66–4.04)	0.286	1.36 (0.83–2.24)	0.223	1.41 (0.92–2.16)	0.115
Student	0.66 (1.87–2.32	0.516	1.05 (0.59–1.86)	0.873	0.92 (0.55–1.53)	0.753
Lower education	1.35 (0.61–3.0)	0.460	0.95 (0.65–1.40)	0.801	1.04 (0.74–1.46)	0.812
Being married	1.09 (0.45–2.63)	0.846	0.80 (0.50–1.30)	0.366	0.91 (0.61–1.38)	0.663

Data is presented as Odd Ratios (95% confidence interval). Estimated values are derived from multivariate regression model including the risk factors presented in the table. Reference groups in the models are: normal BMI, no family history, non-smokers, being employed, higher education level, and unmarried. No significant interaction was detected between age and BMI.

## Data Availability

Data is available upon request.

## Supplementary Materials

The following supporting information can be downloaded at: https://www.mdpi.com/article/10.3390/ijerph192113757/s1, Table S1: Comparative analysis of fasting and non-fasting men and women.

## Abbreviations

Metabolic Syndrome	MetS
Cardiovascular disease	CVD
Type 2 Diabetes mellitus	T2D
Non-communicable diseases	NCDs
United Arab Emirates	UAE
National Cholesterol Education Program Adult Treatment Panel III	NCEP ATP III
Triglycerides	TG
High-density lipoprotein	HDL
Fasting blood glucose	FBG
Glycated hemoglobin A1C	HbA1c
Body mass index	BMI
Odd Ratios	ORs
Confidence interval	CI
Area under the ROC curve	AUC
Likelihood ratio chi-square	LRX^2^
