# Supplementary material for: Metabolic Syndrome in Fasting and Non-Fasting Participants: The UAE Healthy Future Study
Source: Int J Environ Res Public Health. 2022 Oct 22;19(21):13757. doi: 10.3390/ijerph192113757 (PMC9654954; doi:10.3390/ijerph192113757)
Supplement: Supplementary file 1 [file ijerph-19-13757-s001.zip › ijerph-1891221-supplementary.pdf]

Supplementary Table S1: Comparative analysis of fasting and non-fasting men and women.

	Men			Women		
**Continuous (mean)**	**Fasting**	**Non-fasting**	**P value**	**Fasting**	**Non-fasting**	**P value**
Age (years)	24.9	26.9	<0.001	25.1	24.4	0.063
BMI (kg/m^2)	27.5	27.7	0.3446	26.2	25.7	0.2203
Waist (cm)	90.1	91.9	0.0021	79.0	78.3	0.384
HDL (mg/dL)	44.7	43.6	0.0137	54.8	55.9	0.1962
TG (mg/dL)	92.1	127.5	0.00	71.1	80.8	0.0038
Systolic bp (mmHg)	130.9	131.3	0.4908	117.4	117.8	0.5181
Diastolic bp (mmHg)	80.6	80.2	0.295	74.9	74.2	0.1625
FBG	96.4			88.8		
HbA1C (%)	5.27	5.3	0.2459	5.24	5.20	0.3153
**Categorical (%)**						
Smoker	74.6%	52.3%	0.041	4.62%	5.32%	0.641
Family history of heart disease or Metabolic abnormality	55.5%	52.8%	0.185	65.1%	59.0%	0.046
Employed	63.9%	70.2%	<0.001	35.7%	30.2%	0.003
College graduate	46.8%	44.1%	0.217	48.3%	48.1%	0.941
Married	32.7%	45.7%	<0.001	27.1%	19.5%	0.004
P values are derived from t-tests for continuous variables, and chi-square tests for categorical variables.						
